# Pan-cancer analysis of the prevalence and associated factors of lung metastasis and the construction of the lung metastatic classification system

**DOI:** 10.3389/fsurg.2022.922167

**Published:** 2022-07-26

**Authors:** Xiaolong Lv, Lei Yang, Tianyu Liu, Zelin Yang, Chenhao Jia, Huanwen Chen

**Affiliations:** Department of Cardiothoracic Surgery, The First Affiliated Hospital of Chongqing Medical University, Chongqing, China

**Keywords:** lung metastasis, prevalence, associated factors, cancer classification, SEER program

## Abstract

This study first presents an analysis of the prevalence and associated factors of the lung metastasis (LM) database and then uses this analysis to construct an LM classification system. Using cancer patient data gathered from the surveillance, epidemiology, and end results (SEER) database, this study shows that the prevalence of LM is not consistent among different cancers; that is, the prevalence of LM ranges from 0.0013 [brain; 95% confidence interval (95% CI); 0.0010–0.0018] to 0.234 (“other digestive organs”; 95% CI; 0.221–0.249). This study finds that advanced age, poor grade, higher tumor or node stage, and metastases including bone, brain, and liver are positively related to LM occurrence, while female gender, income, marital status, and insured status are negatively related. Then, this study generates four categories from 58 cancer types based on prevalence and influence factors and satisfactorily validates these. This classification system reflects the LM risk of different cancers. It can guide individualized treatment and the management of these synchronous metastatic cancer patients and help clinicians better distribute medical resources.

## Introduction

The lungs are a typical site for the distant metastasis of various types of cancer that significantly worsen patient prognosis (1, 2). It is reported that the survival of lung metastasis (LM) patients may benefit from early diagnosis (3). However, due to the lack of specific signs, many LM patients are underdiagnosed, which may result in the best diagnosis and treatment time window being missed (4). It is well known that effective prediction of LM risk helps clinicians make a rapid diagnosis and provide targeted treatment strategies (5, 6). Many studies have researched the factors associated with LM, attaining results that indicate a range of associated clinical factors, including age, gender, and differentiated grade, which thus provide the basis for LM risk prediction. However, these studies have mainly focused on the primary site of the main cancers (breast cancer, colorectal cancer, liver cancer, etc.) rather than on their metastasis (7–9). Moreover, limited by the relatively small sample size, there are differences among the results gained, which limit their application to clinical practice.

The National Cancer Institute's Surveillance, Epidemiology, and End Results (SEER) program developed in 1973 has recorded dozens of cancer types in millions of cancer patients. This provides the opportunity to investigate the associated factors for LM with relatively high statistical power. In this study, we evaluate the prevalence and associated factors of LM across different cancer types and then construct an LM classification system. This is intended to help clinicians determine individualized management and therapeutic strategies for these cancer patients.

## Materials and methods

### Ethics statement

Cancer is a reportable disease in the United States; the data in the SEER database do not require informed patient consent. This study complied with the Helsinki Declaration in 1964, along with its later amendments and similar ethical standards.

### Study population

All information on the patients included in the present study is derived from the SEER database, which covers about 30% of the total population of the United States. This study included those patients diagnosed with cancer from 2010 to 2016 according to the American Joint Committee on Cancer (AJCC) staging system (7th edition) and excluded those patients who lacked clear LM information or were diagnosed by autopsy or death certificate.

**Figure 1:**
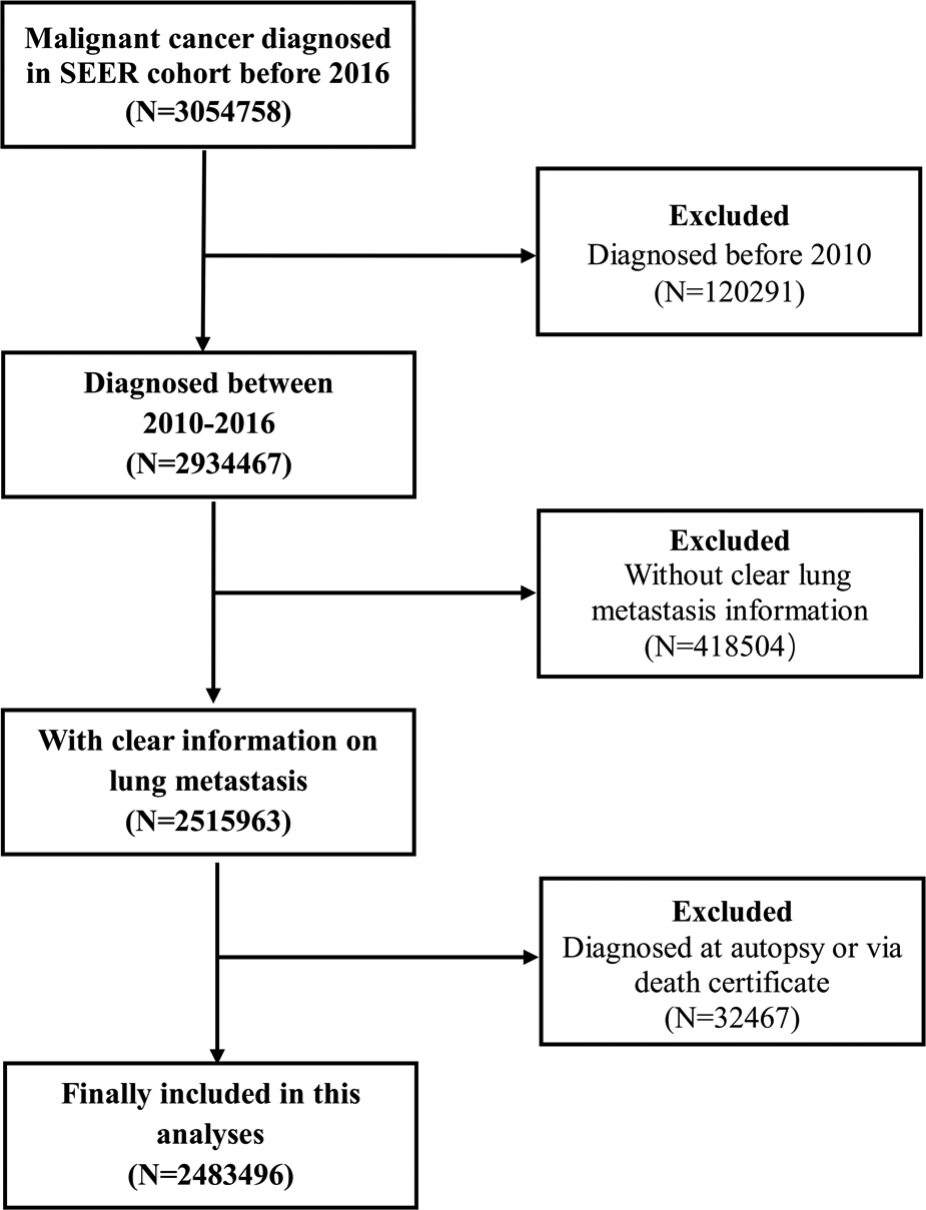
Flowchart showing the patient selection procedure.

Based on these inclusion and exclusion criteria, the patients diagnosed in 2017 were selected as a validation cohort to test the application of the LM classification system. The case listing was generated by SEER*Stat version 8.3.4 (Information Management Service, Inc., Calverton, MD, USA) (Figure 1).

### Statistical analysis

This study uses mean ± standard deviation for quantitative data (on age, etc.), numbers and percentages (*n*, %) for categorical data (sex, etc.), and chi-square and rank-sum tests for the differences between groups. The calculated percentage of a cancer with LM within the total number of cancer patients is taken to describe the prevalence of LM of one cancer. The pooled prevalence of LM for the total population and different races, genders, and diagnosed year subgroups is calculated by a meta-analysis using a random effect model.

A multivariable logistic regression incorporating all factors is applied to determine the associated factors of LM, and the pooled effect sizes for these factors across different cancer types are additionally combined by meta-analysis with a random effect model.

Based on the patient demographic, clinical, and LM features, this study performs an unsupervised hierarchical clustering analysis using the squared Euclidean distance method. The cancers are classified into four categories (A, B, C, and D) according to tree cluster analysis. The multivariable logistic regression model analysis is performed to determine the odds ratio (OR) value of different categories compared with category A, adjusting for the associated factors of age, sex, race, marriage, insurance, and tumor (T) and node (N) stage. The application of this classification system is further validated using the participants diagnosed in the 2017 SEER cohort.

Comprehensive Meta-Analysis (CMA) version 2.0 (Biostat, Englewood, NJ, USA) was used for the meta-analysis, and the Statistical Package for the Social Sciences (SPSS) version 23.0 was used for statistical analysis. Two-tailed *p*-values of <0.05 were considered statistically significant.

## Results

### Participant characteristics

A total of 2, 483,496 cancer patients with 58 cancer types (sites) were included in the research group for this study with 370,318 in the validation group. In the research group, the mean age was 64.26 ± 14.47 years (0–120 years), 49.4% (*n* = 1,227,041) were male, 53.4% were married (*n* = 1,326,816), 80.5% were designated “white” (*n* = 1,998,593), and 81.3% were insured (*n* = 2,018,544); in the validation group, the mean age was 62.94 ± 13.10 years (0–84 years), 49.2% (*n* = 182,248) were male, and 78.8% were white (*n* = 291,938) (Tables 1A,B).

**Table 1A:** Demographic and clinical characteristics distribution of the included patients in the research group.

Factors	Metastatic to lung		Non-metastatic to lung		$\chi^{2}/Z$	*P*
	*N*	%	*N*	%		
All patients	120,070	100.0	2,363,426	100.0		
Age (years)					2,911.1	<0.01
<65	52,156	43.4	1,215,230	51.4		
≥65	67,914	56.6	1,148,196	48.6		
Sex					223.9	<0.01
Male	61,853	51.5	1,165,188	49.3		
Female	58,217	48.5	1,198,238	50.7		
Race					2,155.7	<0.01
White	93,546	77.9	1,905,047	80.6		
Black	15,620	13.0	253,231	10.7		
Asian or Pacific Islander	9,682	8.1	158,690	6.7		
American Indian/Alaska Native	938	0.8	13,974	0.6		
Unknown	284	0.2	32,484	1.4		
Marital status					6,166.3	<0.01
Married	57,047	47.5	1,269,769	53.7		
Unmarried	57,466	47.9	889,011	37.6		
Unknown	5,557	4.6	204,646	8.7		
Insurance status					84.9^a^	<0.01
Uninsured	4,659	3.9	53,346	2.3		
Any medicaid	20,378	17.0	262,634	11.1		
Insured	92,603	77.1	1,925,941	81.5		
Unknown	2,430	2.0	121,505	5.1		
Income					29.1^a^	<0.01
<6000	28,866	24.0	499,507	21.1		
6000–7000	36,204	30.2	686,975	29.1		
7000–8000	17,166	14.3	358,628	15.2		
>8000	37,831	31.5	818,055	34.6		
Differentiated grade					202.3^a^	<0.01
Grade I	3,310	2.8	295,607	12.5		
Grade II	16,718	13.9	690,970	29.2		
Grade III	24,612	20.5	517,582	21.9		
Grade IV	6,228	5.2	108,232	4.6		
Unknown	69,199	57.6	750,986	31.8		
T stage					314.1^a^	<0.01
T1	11,194	9.3	1,030,218	43.6		
T2	16,095	13.4	552,521	23.4		
T3	28,390	23.6	376,785	15.9		
T4	35,611	29.7	166,593	7.0		
Unknown	28,780	24.0	237,309	10.0		
*N* stage					295.3^a^	<0.01
*N*0	36,614	30.5	1,646,143	69.7		
*N*1	24,515	20.4	307,416	13.0		
*N*2	25,434	21.2	185,789	7.9		
*N*3	13,245	11.0	52,774	2.2		
Unknown	20,262	16.9	171,304	7.2		
Bone					176,661.3	<0.01
No	82,565	68.8	2,270,164	96.1		
Yes	33,653	28.0	88,817	3.8		
Unknown	3,852	3.2	4,445	0.2		
Brain					110,685.1	<0.01
No	101,218	84.3	2,325,837	98.4		
Yes	14,166	11.8	33,311	1.4		
Unknown	4,686	3.9	4,287	0.2		
Liver					194,919.2	<0.01
No	77,486	64.5	2,253,525	95.3		
Yes	39,065	32.5	106,056	4.5		
Unknown	3,519	2.9	3,845	0.2		
Cancer site						
Lip	18	0	4,071	0.2		
Tongue	414	0.3	22,067	0.9		
Salivary gland	275	0.2	7,565	0.3		
Floor of mouth	67	0.1	3,225	0.1		
Gum and other mouth	162	0.1	9,178	0.4		
Nasopharynx	166	0.1	3,659	0.2		
Tonsil	232	0.2	13,443	0.6		
Oropharynx	120	0.1	2,771	0.1		
Hypopharynx	177	0.1	3,581	0.2		
Other oral cavity and pharynx	62	0.1	1,259	0.1		
Esophagus	2,470	2.1	23,268	1		
Stomach	2,237	1.9	41,036	1.7		
Small intestine	377	0.3	14,059	0.6		
Colon cancer	7,912	6.6	165,060	7		
Rectum and rectosigmoid junction	4,531	3.8	70,248	3		
Anus, anal canal and anorectum	262	0.2	11,801	0.5		
Liver and intrahepatic bile duct	3,766	3.1	49,685	2.1		
Gallbladder cancer	387	0.3	6,792	0.3		
Other biliary	743	0.6	10,583	0.4		
Pancreas	8,038	6.7	66,151	2.8		
Retroperitoneum	160	0.1	2,200	0.1		
Peritoneum, omentum and mesentery	226	0.2	3,038	0.1		
Other digestive organs	811	0.7	2,644	0.1		
Nose, nasal cavity and middle ear	77	0.1	4,142	0.2		
Larynx	453	0.4	19,082	0.8		
Lung and bronchus	47,105	39.2	281,272	11.9		
Pleura	15	0	143	0		
Trachea, mediastinum and other respiratory organs	126	0.1	899	0		
Bones and joints	629	0.5	4,785	0.2		
Soft tissue including heart	1,807	1.5	19,130	0.8		
Melanoma of the skin	2,690	2.2	139,640	5.9		
Other non-epithelial skin	102	0.1	11,581	0.5		
Breast	7,845	6.5	426,907	18.1		
Cervix uteri	995	0.8	21,707	0.9		
Corpus and uterus, NOS	2,619	2.2	90,281	3.8		
Ovary	2,233	1.9	35,503	1.5		
Vagina	111	0.1	2,222	0.1		
Vulva	130	0.1	8,356	0.4		
Other female genital organs	408	0.3	4,702	0.2		
Prostate	2,410	2	342,300	14.5		
Testis	1,262	1.1	15,836	0.7		
Penis	57	0	2,421	0.1		
Urinary bladder	1,942	1.6	121,387	5.1		
Kidney and renal pelvis	8,480	7.1	92,963	3.9		
Ureter	126	0.1	3,119	0.1		
Other urinary organs	136	0.1	1,852	0.1		
Eye and orbit	39	0	5,209	0.2		
Brain	47	0	35,235	1.5		
Cranial nerves other nervous system	17	0	2,287	0.1		
Thyroid	1,270	1.1	87,717	3.7		
Other endocrine including thymus	503	0.4	3,949	0.2		
Hodgkin lymphoma	74	0.1	1,945	0.1		
Non-Hodgkin lymphoma	513	0.4	21,555	0.9		
Myeloma	61	0.1	1,550	0.1		
Leukemia	114	0.1	3,253	0.1		
Mesothelioma	357	0.3	4,834	0.2		
Kaposi sarcoma	18	0	352	0		
Miscellaneous	1,686	1.4	7,926	0.3		

^a^ Ordinal categorical variables were compared using the rank-sum test.

**Table 1B:** Demographic and clinical characteristics distribution of the included patients in the validation group.

Factors	Metastatic to lung		Non-metastatic to lung		$\chi^{2}/Z$	*P*
	*N*	%	*N*	%		
All patients	18,571	100.0	351,747	100.0		
Age (years)						
<65	7,776	41.9	174,422	49.6	420.1	<0.01
≥65	10,795	58.1	177,325	50.4		
Sex					31.8	<0.01
Male	9,514	51.2	172,734	49.1		
Female	9,057	48.8	179,013	50.9		
Race					359.2	<0.01
White	14,286	76.9	277,652	78.9		
Black	2,365	12.7	37,896	10.8		
Asian or Pacific Islander	1,627	8.8	26,104	7.4		
American Indian/Alaska Native	176	0.6	2,161	0.6		
Unknown	8,051	2.2	7,934	2.3		
Differentiated grade					83.5^a^	<0.01
Grade I	482	2.6	49,918	14.2		
Grade II	2,473	13.3	102,909	29.3		
Grade III	3,363	18.1	63,848	18.2		
Grade IV	1,017	5.5	17,971	5.1		
Unknown	11,236	60.5	117,101	33.3		
T stage					110.5^a^	<0.01
T1	1,440	7.8	141,830	40.3		
T2	2,744	14.8	77,830	22.1		
T3	3,513	18.9	53,663	15.3		
T4	5,503	29.6	23,331	6.6		
Unknown	5,371	28.9	55,093	15.7		
N stage					100.5^a^	<0.01
N0	5,461	29.4	231,027	65.7		
N1	3,538	19.1	44,566	12.7		
N2	3,527	19.0	26,400	7.5		
N3	2,247	12.1	7,826	2.2		
Unknown	3,798	20.5	41,928	11.9		
Bone					24,921.6	<0.01
No	12,684	68.3	336,313	95.6		
Yes	5,482	29..5	14,965	4.3		
Unknown	405	2.2	469	0.1		
Brain						
No	15,776	84.9	346,034	98.4	15,267.0	<0.01
Yes	2,287	12.3	5,305	1.5		
Unknown	508	2.7	408	0.1		
Liver					26,384.5	<0.01
No	12,344	66.5	335,327	95.3		
Yes	5,832	31.4	16,003	4.5		
Unknown	395	2.1	417	0.1		
Cancer site						
Lip	0	0.0	514	0.1		
Tongue	58	0.3	3,612	1.0		
Salivary gland	42	0.2	1,145	0.3		
Floor of mouth	7	0.0	397	0.1		
Gum and other mouth	20	0.1	1,398	0.4		
Nasopharynx	17	0.1	532	0.2		
Tonsil	58	0.3	3,612	1.0		
Oropharynx	25	0.1	471	0.1		
Hypopharynx	27	0.1	494	0.1		
Other oral cavity and pharynx	6	0.0	216	0.1		
Esophagus	411	2.2	3,524	1.0		
Stomach	333	1.8	6,309	1.8		
Small intestine	64	0.3	2,301	0.7		
Colon cancer	836	4.5	16,860	4.8		
Rectum and rectosigmoid junction	564	3.0	8,171	2.3		
Anus, anal canal and anorectum	45	0.2	1,813	0.5		
Liver and intrahepatic bile duct	741	4.0	9,781	2.8		
Gallbladder cancer	78	0.4	1,011	0.3		
Other biliary	113	.0.6	1,661	0.5		
Pancreas	1,358	7.3	10,831	3.1		
Retroperitoneum	22	0.1	302	0.1		
Peritoneum, omentum and mesentery	39	0.2	447	0.1		
Other digestive Organs	151	0.8	475	0.1		
Nose, nasal cavity, and middle ear	14	0.1	638	0.2		
Larynx	53	0.3	2,636	0.7		
Lung and bronchus	6,980	37.6	42,052	12.0		
Pleura	2	0.0	9	0.0		
Trachea, mediastinum, and other respiratory organs	17	0.1	120	0.0		
Bones and joints	106	0.6	697	0.2		
Soft tissue including heart	307	1.7	2,941	0.8		
Melanoma of the skin	431	2.3	22,139	6.3		
Other Non-epithelial skin	14	0.1	1,839	0.5		
Breast	1,191	6.4	66,299	18.8		
Cervix uteri	162	0.9	3,243	0.9		
Corpus and uterus, NOS	482	2.6	14,636	4.2		
Ovary	315	1.7	4,921	1.7		
Vagina	19	0.1	344	0.1		
Vulva	21	0.1	1,372	0.4		
Other female genital organs	86	0.5	995	0.3		
Prostate	408	2.2	52,192	14.8		
Testis	195	1.0	2,489	0.7		
Penis	11	0.1	378	0.1		
Urinary bladder	298	1.6	17,935	5.1		
Kidney and renal pelvis	1,436	7.7	15,079	4.3		
Ureter	19	0.1	472	0.1		
Other urinary organs	23	0.1	292	0.1		
Eye and orbit	3	0.0	743	0.2		
Brain	4	0.0	5,122	0.5		
Cranial nerves other nervous system	3	0.0	333	0.1		
Thyroid	214	1.2	12,585	3.6		
Other endocrine including thymus	77	0.4	578	0.2		
Hodgkin lymphoma	56	0.3	974	0.3		
Non-Hodgkin lymphoma	5	0.0	157	0.1		
Myeloma	0	0.0	0	0.0		
Leukemia	0	0.0	0	0.0		
Mesothelioma	58	0.3	700	0.2		
Kaposi sarcoma	11	0.1	254	0.1		
Miscellaneous	549	3.0	2,102	0.6		

^a^ Ordinal categorical variables were compared using the rank-sum test.

### Prevalence of LM

The prevalence of LM was not consistent among different cancers. It ranged from 0.0013 [brain; 95% confidence interval (CI); 0.0010–0.0018] to 0.234 (“other digestive organs”; 95% CI; 0.221–0.249). Based on the meta-analysis, the pooled prevalence of LM was 0.037 (95% CI; 0.029–0.047). Subgroup analysis showed the pooled LM prevalence in males and females as 0.038 (95% CI; 0.030–0.049) and 0.037 (95% CI; 0.029–0.047), respectively, with no statistically significant difference (*p* = 0.849).

**Figure 2:**
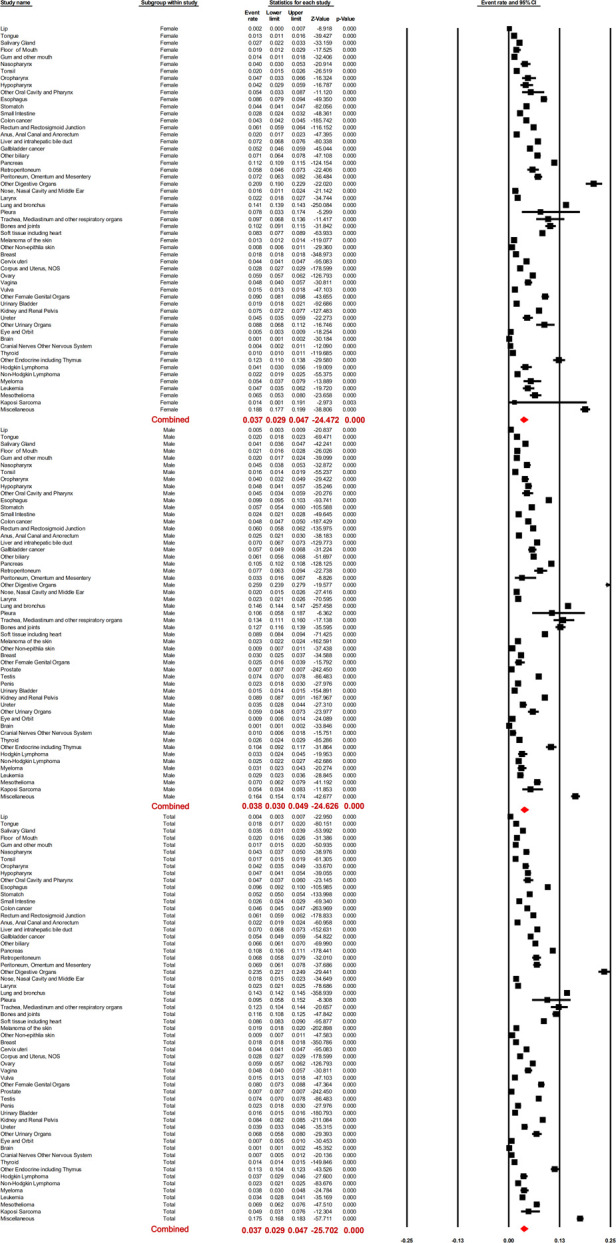
Forest plot for the prevalence of lung metastases for different cancer sites, the pooled prevalence of male and female patients, and the total population with random effects mode.

**Figure 3:**
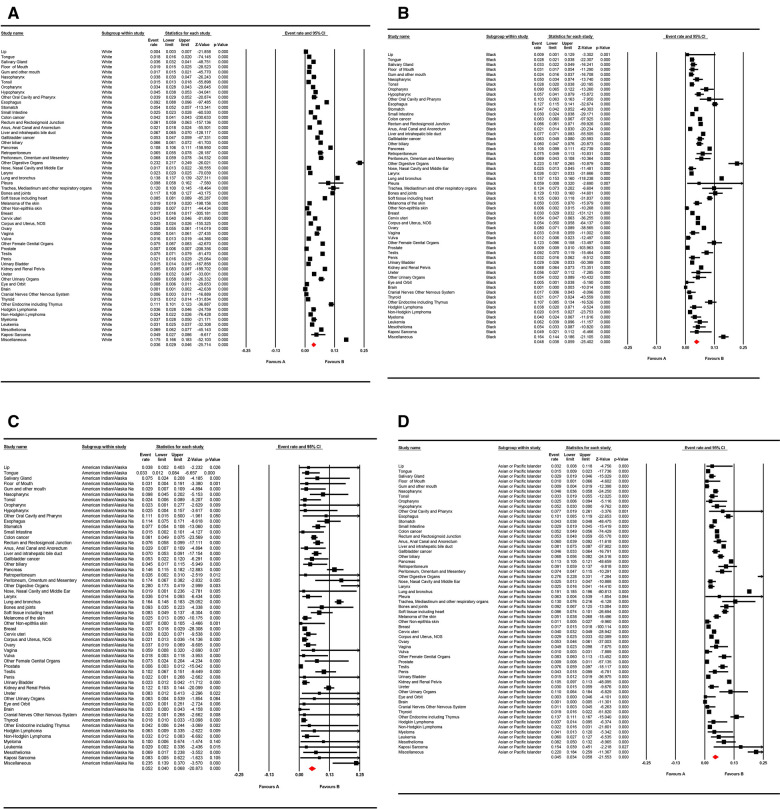
Forest plot for the pooled prevalence of lung metastases for different cancer sites in (**A**) “white,” (**B**) “black,” (**C**) American Indian/Alaska Native population, and (**D**) Asian or Pacific Islander populations with random effects mode.

**Figure 4:**
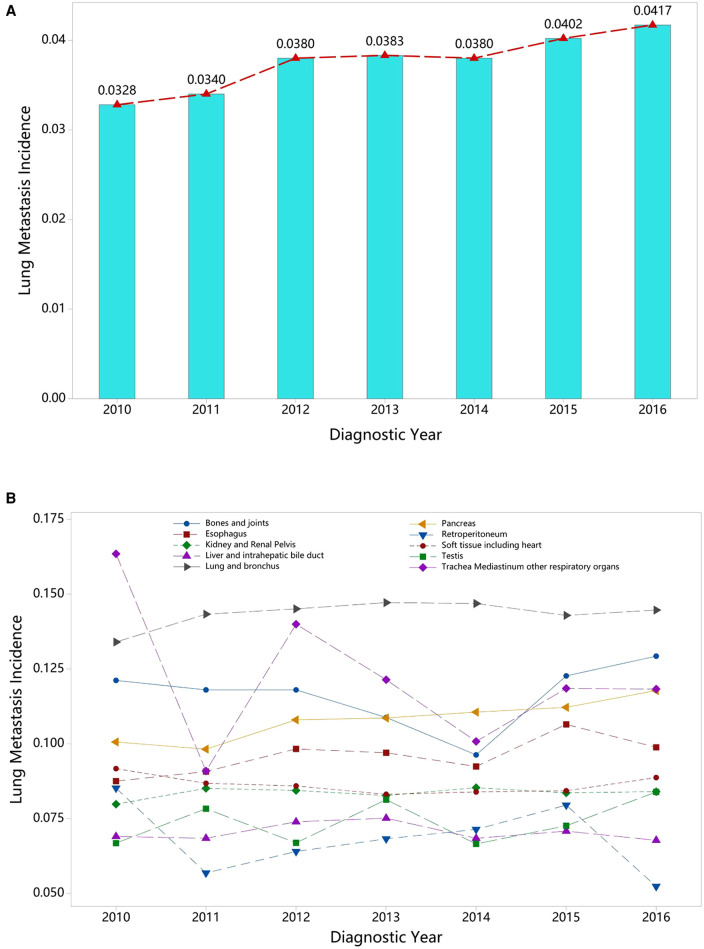
Annual variation curve of (**A**) the pooled prevalence of LM from 2010 to 2016 and (**B**) the top ten of pooled prevalence LM across 58 cancer sites (excluding mixed cancer sites and cancers with a sample of less than 100).

With respect to different race groups, the pooled prevalence of LM in the white, Asian, and Pacific Islander, African-origin (“black”), and American Indian/Alaska Native groups was 0.036 (95% CI; 0.029–0.045), 0.045 (95% CI; 0.034–0.058), 0.048 (95% CI; 0.038–0.059), and 0.052 (95% CI; 0.040–0.068), respectively. The pooled LM prevalence gradually increased according to the year of diagnosis, from 0.0328 in 2010 to 0.0417 in 2016 (Figures 2–4A). The top ten LM prevalence cancer sites remained fairly steady over the period studied; they were cancer of the trachea, mediastinum, and other respiratory organs, lung and bronchus, bones and joints, pancreas, soft tissue (including heart), esophagus, retroperitoneum, kidney and renal pelvis, liver and intrahepatic bile duct, and testis (Figure 4B).

### Associated factors of LM occurrence

**Figure 5:**
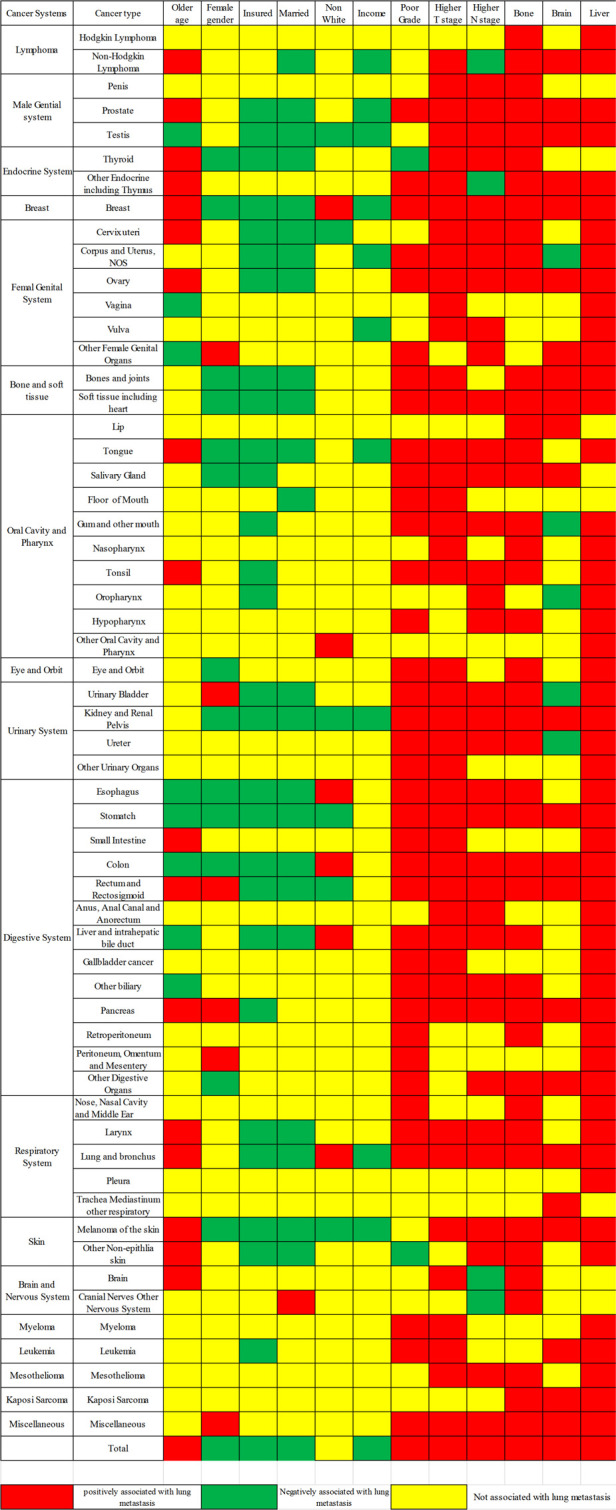
Lung metastasis risk factors for the 58 metastatic cancer types; red and green show risk and protective factors, respectively, in the LM of cancers, while yellow indicates factors that were not significant.

The multivariable logistic regression analysis showed that advanced age, poor grade, higher T or N stage, and metastases including bone, brain, and liver were positively related to LM occurrence, while female gender, income, marital status, and insured status were negatively related. However, these associations were not consistent across different cancer types (Figure 5). Based on the meta-analysis, the pooled ORs were as follows: age 1.126 (95% CI; 1.050–1.209), grade 1.074 (95% CI; 1.055–1.093), T stage 1.150 (95% CI; 1.124–1.176), and N stage 1.067 (95% CI; 1.0049–1.085); bone 1.525 (95% CI; 1.422–1.637), brain 1.100 (95% CI; 1.038–1.166), and liver 1.955 (95% CI; 1.783–2.143); and gender 0.921 (95% CI; 0.867–0.978), marital status 0.954 (95% CI; 0.942–0.966), insured status 0.877 (95% CI; 0.858–0.897), race 0.955 (95% CI; 0.943–0.966), and income 0.977 (95% CI; 0.967–0.986). These results show age, grade, higher T or N stage, metastases in bone, brain, and liver to be risk factors for LM risk, while gender, marital status, insured status, race, and income were protective factors.

### Prevalence and influence factor-based cancer classification

**Figure 6:**
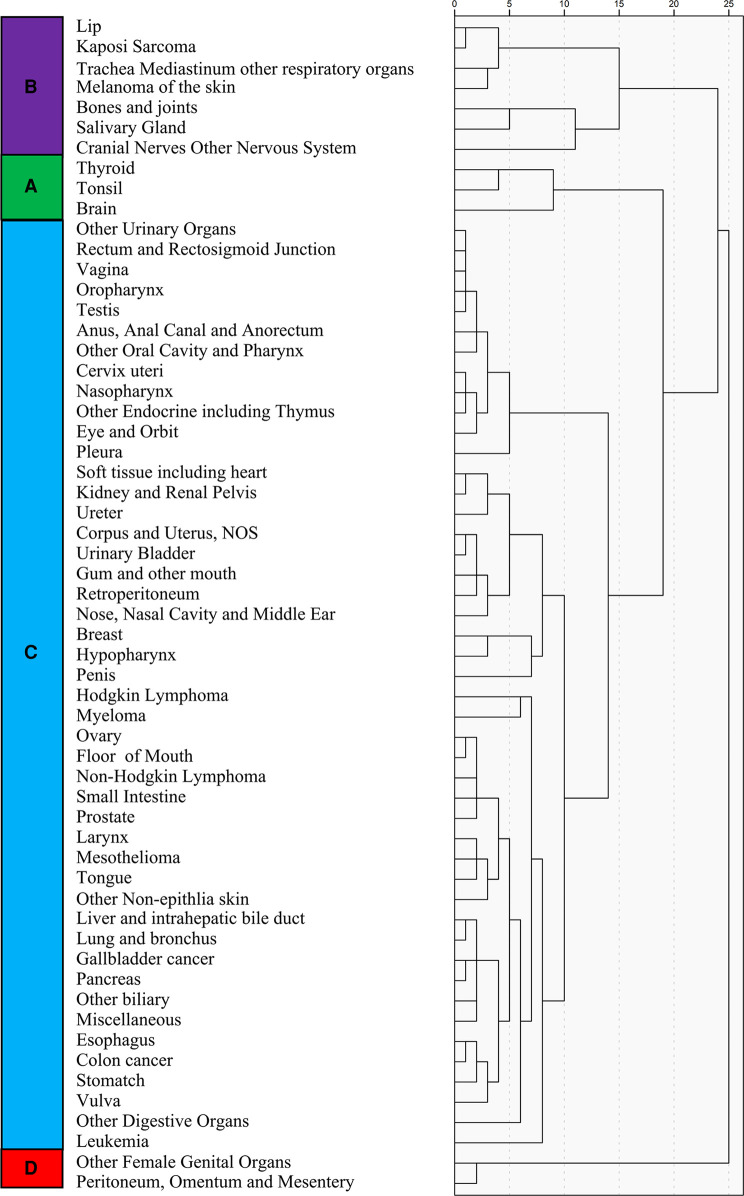
Unsupervised hierarchical cluster analysis for the classification of cancer types; the 58 cancer types are sub-grouped into four categories (**A–D**).

The 58 cancer types were classified into four main subgroups, namely, categories A, B, C, and D, based on unsupervised hierarchical clustering analysis (Figure 6). Significant differences were found among these four categories (chi-square value: 7,344.16, *p* < 0.01). Category D, which included other female genital organs, peritoneum, omentum, and mesentery, had the highest LM prevalence (0.079), while category A, including thyroid, tonsil, and brain, had the lowest (0.021).

**Figure 7:**
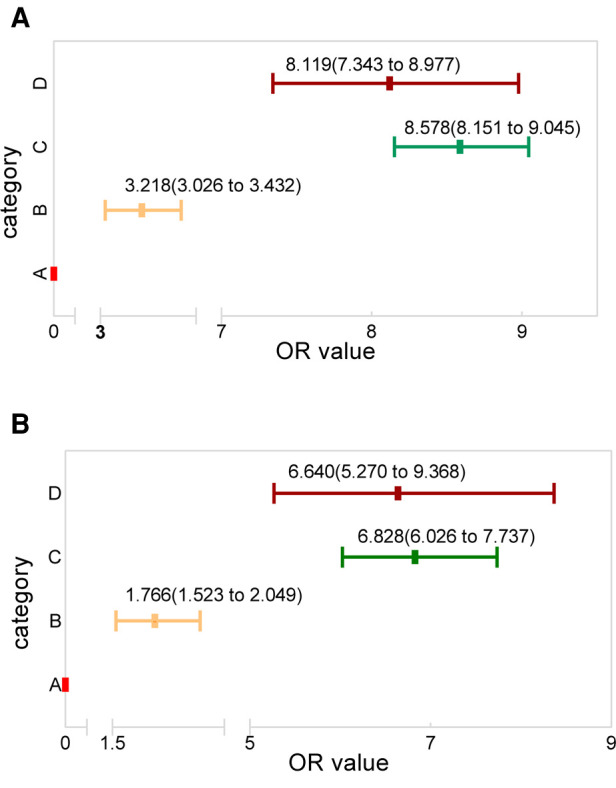
The OR value distribution among the four categories, with category A as reference for the (**A**) research and (**B**) validation groups.

Using category A as the reference, the regression analysis showed that the OR value for categories B, C, and D was 3.218 (95% CI; 3.026–3.432), 8.578 (95% CI; 8.151–9.045), and 8.119 (95% CI; 7.343–8.977), respectively (Figure 7A). The classification system was validated using the participants diagnosed in the 2017 SEER cohort. These results also showed category A to have a significantly lower risk of LM occurrence than categories B (1.766; 95% CI; 1.532–2.049), C (6.828; 95% CI; 6.026–7.737), and D (6.640; 95% CI; 5.270–9.368) (Figure 7B).

## Discussion

Utilizing the SEER database from 2010 to 2016, this study evaluated the prevalence and risk factors of LM in various cancers and built a cancer classification system based on the information gained.

The prevalence of LM in the different cancers types varied significantly, ranging from 0.0013 (brain; 95% CI; 0.0010–0.0018) to 0.234 (other digestive organs; 95% CI; 0.221–0.249), while interestingly, the top ten for LM prevalence remained very similar over the period studied and fluctuated over a limited range. In the stratified racial subgroup analysis, the prevalence of LM ranged between 0.036 (white; 95% CI; 0.029–0.045) and 0.052 (American Indian/Alaska Native; 95% CI; 0.040–0.068). This is consistent with previous reports, in which a greater proportion of African American patients than Caucasian or Asian people have presented with metastatic disease (10). That people of African origin (blacks) continue to be diagnosed with more advanced cancers may partly explain this difference (11). Thus, metastatic screening is vital for this group.

On the other hand, the pooled prevalence of LM increased during the period studied, rising from 0.328 in 2010 to 0.412 in 2016, and the majority of patients were at an advanced stage at the time of diagnosis, which generally indicated the need for prompt metastatic screening.

This study analyzed nearly all the risk factors of LM across various cancer types using a vast population from the SEER database for the first time. It found older age, poor grade, higher T or N stage, and metastases in the bone, brain, and liver to be positively related to LM; female gender, marital and insured status, and income were negatively related to LM, and non-white race was not associated with LM on the whole. Most of the risk factors considered for specified cancers have been reported previously, and the results here were consistent (4, 8, 12, 13).

Some other risk factors have been reported. For example, extrathyroidal extension has been identified as an independent risk factor in thyroid cancer patients with LM (14); elevated alpha-fetoprotein is relevant to a higher risk of LM in hepatocellular carcinoma (9); and special (neither squamous nor adenocarcinoma) histological types have been correlated with a higher risk for LM in cervical cancer (15). These factors reported were only correlated with one particular cancer type, but all were common to all cancers in the present study. In addition, the present study found that the associations between these risk factors and LM were not consistent across cancer types, indicating that these risk factors are both homogeneous and heterogeneous. Thus, studies identifying specific LM risk factors should be performed involving the factors that were a basis for the cancer classification system in the present study.

Previous studies have reported that early detection of metastasis can benefit patients as it may lead (1) to the early diagnosis, management, and prediction of progression; (2) avoidance of highly toxic therapies; and (3) improvements in the quality of life after therapy (16–18). Thus, the early detection and timely treatment of metastasis is necessary. The use of biomarkers and genes has been chosen as a potential approach to screening and early clinical detection (19), but the huge cost, complex process, and long detection period have limited clinical application. Therefore, anatomical locations can be used for prediction on the basis that similar anatomic structures make similar metastases (13). However, this study has shown that the anatomical hypothesis can only partly explain the fact that different tumor locations make different metastatic sites and also that the prevalence of LM is inconstant in different cancers of the same system.

For lung cancer, imaging remains the most reliable and generally accepted method to screen for early diagnosis, but it has some limitations, such as radiation exposure and high cost (20). The present study has constructed a cancer classification system based on LM prevalence and risk factor to predict LM. Compared with imaging, therefore, prediction using this classification system could avoid unnecessary radiation exposure and cost. If someone has been identified as having high-risk factors of LM based on this system, physicians could employ the imaging approach to diagnose early and then develop individualized management strategies in advance, leading to a better prognosis for patients.

The present study also conducted a verification of the cancer classification system developed with the 2017 SEER data, and it remained reliable. This confirms that the cancer classification system developed here could help clinical decision-making through its satisfactory predictive abilities. It would be reasonable to closely monitor LM patients, mainly by regular chest computed tomography (CT), in order to regulate their psychological states, since waiting for treatment is known to cause anxiety, distress, and uncertainty (21). Also, prophylactic treatment might be advised since cancer treatment can come with high costs and toxicity. Patients could probably benefit more from preventive treatment, (22), and their families could enjoy more accurate treatment expectations and avoid doctor–patient conflicts. Similarly, medical resource distribution and medical insurance choices have become a global concern, (23), and in this context, this LM classification system could help clinicians better manage and distribute medical resources for these cancer patients since medical resource distribution varies for cancers with different risks. For example, clinicians could shorten the interval of monitor, which could help in early diagnosis of LM for these patients and in carrying out preventive treatment for these high-risk group patients.

This study has some limitations. First, asymptomatic LM patients and those patients who developed LM later, during the initial cancer course, were not included in the SEER database. Thus, the prevalence of LM determined here may be an underestimate, and more studies are needed to (further) confirm these results. Second, the random-effect model was used in the meta-analysis because of the significant heterogeneity among cancers; therefore, the combined prevalences were relatively conservative. Third, some cancer data were combined in the SEER database, such as the grouping “other digestive organs,” which may have biased the clustering analysis; therefore, the present study excluded these prevalences from the analysis of trends (change over time). Fourth, some information that may influence LM, such as lung function and performance and smoking status, was not included in the SEER database. Finally, the cancer classification system developed lacks further validation; therefore, we feel that the feasibility of the classification system can be further verified by expanding the amount of data or using external databases in the future.

## Conclusion

This study has evaluated the prevalence of LM in various cancers, determined that the prevalence of LM was inconsistent in them, and found that the pooled prevalence showed an increasing trend from 2010 to 2016. In addition, a series of LM risk factors have been identified (older age, poor grade, and higher T or N stage), metastases have been positively associated with LM, while the risk factors in various cancer types have been shown to be inconsistent. According to the LM prevalence and risk factors, the cancers were divided into four groups. The different groups in the classification reflect the risk of LM and may help physicians to predict the occurrence of LM and conduct individualized management strategies for high-risk LM cancer patients.

## Data Availability

The original contributions presented in the study are included in the article/Supplementary Material, and further inquiries can be directed to the corresponding author/s.
